# Purification and Characterization of Glutathione Binding Protein GsiB from* Escherichia coli*

**DOI:** 10.1155/2018/3429569

**Published:** 2018-11-01

**Authors:** Zhongshan Wang, Xiaokun Xia, Meixian Zhang, Jiawei Fang, Yanqiang Li, Meng Zhang

**Affiliations:** ^1^Jiangsu Province Key Laboratory of Anesthesiology, Xuzhou Medical University, Xuzhou, China; ^2^Department of Gynecology, Central Hospital of Xuzhou, Affiliated Hospital of Southeast University, Xuzhou, China; ^3^Xuzhou Medical University, China

## Abstract

**Objectives:**

To purify and characterize the glutathione binding protein GsiB of glutathione importer (GSI) in* Escherichia coli (E. coli)*.

**Results:**

The coding sequence of GsiB was cloned from* E. coli *MG1655 and expressed in BL21(DE3). GsiB protein was expressed and purified to homogeneity using Ni-affinity and gel filtration chromatography. SDS-PAGE of purified GsiB showed a single protein band of molecular mass 56 kDa, while native gel showed two bands around 56 kDa and 110 kDa. Gene knockout showed that GsiB was essential for GSI mediated glutathione import. Interactions of GsiA, B, C, and D were determined using bacterial two-hybrid method. Without glutathione, GsiB showed no direct interaction with the other three proteins. However, GsiB could interact with GsiC and GsiD when using glutathione as sole sulfur source.

**Conclusions:**

GsiB functions in* E. coli* was characterized which could help elucidate the glutathione import mechanism in gram-negative bacteria.

## 1. **Introduction**

Glutathione (*γ*-L-glutamyl-L-cysteinyl-glycine; GSH) is the most important endogenous antioxidant across all kingdoms of life. GSH is a tripeptide consisting of glutamate, cysteine, and glycine amino acids. GSH is distributed ubiquitously and usually attains mM concentrations in human body. It plays an important role in maintaining the intracellular redox homeostasis [1], as well as in the detoxification of xenobiotics and their metabolites [2, 3]. GSH also functions in salvage of cysteine [4] and cell signaling [5, 6]. In cell, glutathione manifests predominantly in thiol-reduced form (GSH) [7]. A small quantity of glutathione is in oxidation form, such as glutathione disulfide (GSSG) or disulfides with target proteins [8]. The intracellular GSH homeostasis can be maintained by different pathways, including de-novo synthesis, GSH redox cycling, and direct uptake [9]. GSH import by bacteria may serve as organic sulfur resource [10–12]. However, the mechanisms that underpin glutathione uptake still need further investigation.

The specific glutathione importer (GSI) in bacteria was identified in 2005 [10]. This importer consists of* GsiA*,* -B*,* -C*, and*-D*, which encodes ATP binding protein, glutathione binding protein, and two inner-membrane components [13]. The specific glutathione recognition is mediated by GsiB and import is ATP dependent [10, 14].

Herein, the spectrum of expression, purification and characterization of* GsiB *from* E. coli* was described. The* in vitro* and* in vivo* functions of GisB were investigated. Studies of GsiB will help to clarify the mechanism of specific glutathione import in bacteria.

## 2. Materials and Methods

### 2.1. Strains, Plasmids and Chemicals

The* E. coli *strains MG1655, BL21 (DE3), DH5*α*, and plasmid plou3 were preserved in our laboratory. Vectors pET11a-link-NGFP, pMRBAD-link-CGFP, pN-Z, and pC-Z were gifts presented by Professor Lynne Regan of Yale University.* Pfu* polymerase and T4 DNA ligase and restriction enzymes were purchased from NEB. Other chemicals were purchased from Sangon Biotech.

### 2.2. Heterologous Expression of GisB Protein

*GsiB *gene (accession number: HM217135) was amplified from genomic DNA of* E. coli* MG1655 using primers GsiB-F and GsiB-R (Table 1). The gene was cloned into expression plasmid plou3 and transformed into BL21 (DE3). MBP (Maltose binding protein) was used as fusion prtoein. BL21 (DE3) was grown in Luria-Bertani (LB) medium (containing ampicillin 100 *μ*g/ml) at 37°C till OD_600_ reach about 0.5-0.6. Protein expression was induced by adding 0.1 mM IPTG and grown at 22°C for 20 h. Cells were harvested by centrifugation at 5000 g for 15 min at 4°C.

### 2.3. Purification of GsiB Protein

The cell pellet was resuspended in 50 mM Tris/HCl buffer (pH 7.5) containing 100 mM NaCl, 1 mM phenylmethanesulfonyl fluoride (PMSF), 1 *μ*M lysozyme lysozyme, and 1 *μ*M DNaseI. The cell was disrupted by using homogenizer FB-110X (LiTu, China) with 800 MPa. The sample was centrifuged at 8,000 g for 15 min and supernatant was loaded onto Ni^2+^ affinity column (GE Healthcare). The protein was washed with 50 mM Tris/HCl pH 7.5, 300 mM NaCl, and 30 mM imidazole. GsiB-MBP was eluted with 50 mM Tris/HCl pH 7.5, 300 mM NaCl, and 300 mM imidazole. Imidazole was removed by desalting. The protein was then digested with TEV protease and MBP was removed by MBP column. GsiB was further purified using Superdex 200 column (GE Healthcare) (buffer contains 50 mM Tris/HCl pH 7.5, 300 mM NaCl, and 5 % (v/v) glycerol). The purity of GisB was determined by SDS-PAGE. Native gel was performed to analyze protein conformations. The protein was concentrated to 5 mg/ml, which was measured by Nanodrop 2000 (Thermo Scientific).

### 2.4. Western Blot Analysis

Westerrn blot was carried out with anti-6×His monoclonal antibody (Abcam, anti-His, 400 *μ*g/ml, 1:1000 (v/v)) [15] and horseradish peroxidase labeled antibody (Abcam, goat antimouse, 0.8 mg/ml, 1:5000 (v/v)).

### 2.5. GsiB Interacts with Other Components

GFP fragments reassembly protocol was used to determine interaction of GsiB with other GSI components [16, 17].* GsiA*,* GsiB*,* GsiC*, and* GsiD* were cloned into pMRBAD-link-CGFP and pET11a-link-NGFP (With primers in Table 1). Any two recombinant plasmids carrying N- and C-fragment of GFP were simultaneously transformed into BL21(DE3) with 10 ng of DNA. The recombinant cells were plated onto selective agar medium, containing kanamycin (35 *μ*g/ml) and ampicillin (100 *μ*g/ml). Single colonies were selected and incubated with appropriate antibiotic. Fresh overnight culture was diluted (1:1000 (v/v)) and 100 *μ*l medium was plated onto screening medium, containing 0.2% arabinose and 10 *μ*M IPTG. The plates were incubated at 20°C for 2 days to induce protein expression and interacion.

The M9 medium plate [18] was also made with MgSO_4_ replaced by MgCl_2_. Reduced glutathione (1 mM, ≥98%) was added as only sulfur source to characterize the interaction.

### 2.6. Characterization of Glutathione Binding Activity of GsiB

The glutathione binding activity of GsiB was determined by native gel. Purified GsiB was incubated with 5 mM GSH or GSSG and separated on 12% Native-PAGE. The native gel electrophoresis was performed basing on theoretical pI 8.22 of GsiB. The protein samples incubated with glutathione were also analyzed by 12% SDS-PAGE.

### 2.7. GsiB In-Vivo Function Assay

It was supposed that gram-negative bacteria uptake glutathione mainly through *γ*-glutamyltranspeptidase (GGT) pathway or GSI complex [10]. The* GsiB* and* ggt* gene of* E. coli* MG1655 were deleted with *λ*Red recombination system [19, 20]. The kanamycin resistant DNA was amplified from pKD4 with primers GsiB del-F and GsiB del-R (Table 1). The PCR product had 58 bp upstream and 58 bp downstream homologous to adjacent sequence of* GsiB*, which was digested with* Dpn*I and gel-purified. pKD46 was transformed into* E. coli* MG1655 by CaCl_2_ method. The cell with pKD46 was grown in SOB medium at 30°C to an OD_600_ of around 0.5. 2 mM L-arabinose was added 1 h before cell collection. Competent cell was made by washing with 10% glycerol. 50 ng of PCR product was mixed with 50 *μ*l of competent cell. Electroporation was performed using MicroPulser (Bio-Rad) with a 0.1 cm chamber.

*ggt* gene was deleted as above. The chloramphenicol fragment was amplified with 60 bp upstream and 60 bp downstream homologous to adjacent regions of* ggt *(primer GGT del-F and GGT del-R) (Table 1).* GsiB* and* ggt* gene deletion was verified by PCR with primers Del-F and GsiB-Del-R or GGT-Del-R (Table 1).

The cell growth and glutathione uptake curves of mutant strains were measured. M9 minimal medium [18] was used with MgSO_4_ replaced by MgCl_2_. Glutathione (1 mM, ≥98%) was served as the only sulfur source.* GsiB* was cloned into pBAD24 (primers GsiB-F and GsiB-R) and transformed into mutant strains to compensate for gene defection.

## 3. Results

### 3.1. Expression and Purification of GsiB

**Figure 1:**
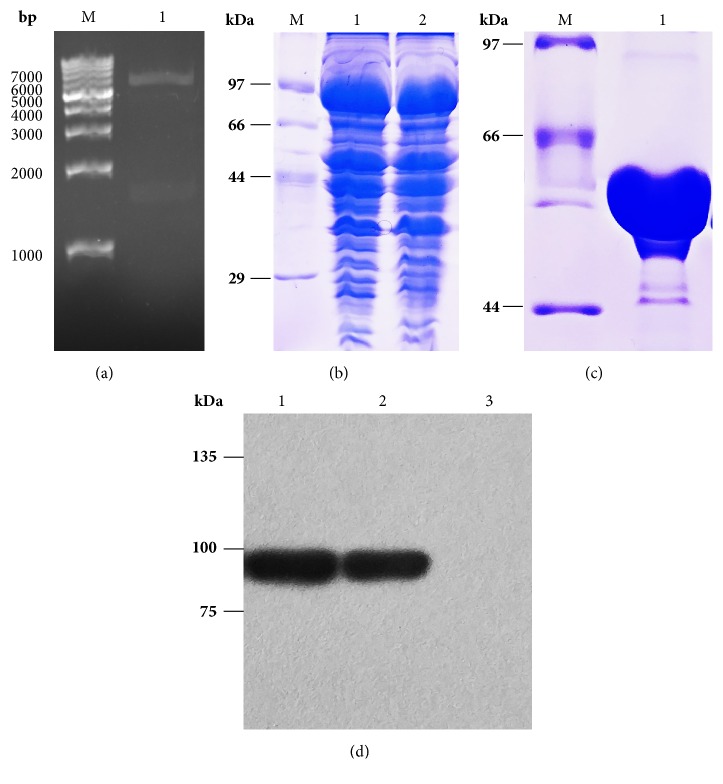
Expression and purification of GsiB from* E.coli*. (a) Recombinant plasmid digestion with restriction enzymes. M: Marker; Lane 1: plasmid digested with* Nco*I and* Hind*III. (b) SDS-PAGE analysis of GsiB expression. M: Marker; Lanes 1-2: total protein and soluble fraction of GsiB induced with 0.1 mM IPTG at 22°C for 20 h. (c) Purity analysis of GsiB. The protein was separated on 12 % (v/v) SDS-PAGE and analyzed with QuantiyOne software. M: Marker; Lane 1: purified GsiB protein; (d) Western blot analysis. Lanes 1-2: total protein and soluble fraction of GsiB in BL21 (DE3) grown at 22°C for 20 h induced with 0.1 mM IPTG; Lane 3: total protein of GsiB in BL21 (DE3) grown at 22°C for 20 h without IPTG.

The* GsiB* gene was amplified from* E. coli MG1655 *genome and cloned into plou3 vector which was derived from pMAL-c2X. A 6×His tag and a TEV protease cleavage site were added before and behind MBP to facilitate protein purification. The resultant plasmid was denominated plou3-gsiB (Figure 1(a)). The insertion of* GsiB* gene was confirmed by DNA sequencing.

GsiB was expressed in BL21(DE3) and expression condition was optimized. Induction with 0.1 mM IPTG at 22°C for 20 h will give high productivity of soluble GsiB (Figure 1(b)). GsiB-MBP fusion protein was firstly purified by Ni^2+^ column and then digested with TEV protease. The MBP was removed by Ni^2+^ and MBP column. The protein was further purified by gel filtration. 12% SDS-PAGE analysis showed that the molecular mass of GsiB was about 56 kDa with purity in excess of 90% (Figure 1(c)). The protein was concentrated to 5 mg/ml and used for glutathione binding activity assay. Approximately 0.8 mg of GsiB protein was obtained from per liter of LB medium.

Western blot confirmed the expression of GisB. As there was a 6×His tag at the N terminal of GsiB-MBP, anti-6×His antibody was used here (Figure 1(d)).

### 3.2. Characterization of Glutathione Binding Activity of GsiB

**Figure 2:**
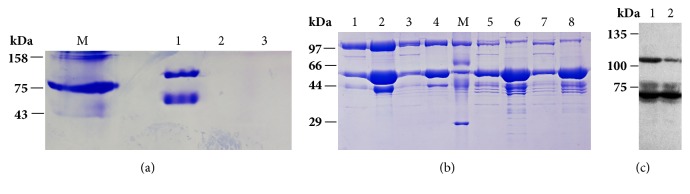
Native gel and SDS-PAGE analysis of GsiB binding activity with GSH and GSSG. (a) Native gel analysis of GsiB protein with GSH and GSSG. M: Marker; Lane 1: purified GsiB protein; Lane 2: GsiB incubated with GSH; Lane 3: GsiB incubated with GSSG. (b) SDS-PAGE analysis of GsiB protein with GSH and GSSG. M: Marker; Lanes 1-4: GsiB protein incubated at 25°C for 2 h; Lane 5-7: same protein aliquots as Lanes 1-4 incubated with GSH (Lanes 5-6) and GSSG (Lane 7-8). Lanes 1, 3, 5, and 7 were GsiB stored in -80°C for 6 months. Lanes 2, 4, 6, and 8 were freshly purified GsiB. (c) Western blot analysis of purified GsiB. Lane 1: purified GsiB protein; Lane 2: GsiB protein with GSH.

The purified GsiB was shown to have two different conformations in native gel. GsiB protein might be present as monomer and dimer (Figure 2(a)). GsiB protein was incubated with reduced (GSH, ≥98%) and oxidized (GSSG, ≥98%) glutathione at 25°C for 2 h. However, after incubation with GSH or GSSG, there was no protein band that could be detected in native gel (Figure 2(a)). This phenomenon might be explained by GsiB conformational change, caused by binding GSH or GSSG. To confirm if the protein was degraded in Figure 2(a), SDS-PAGE was performed. The result showed that GsiB protein was not degraded (Figure 2(b)). The conformational change might confer change of surface charge of GsiB protein. As reversing of cathode and anode position showed protein band in native gel (data not shown). However, the protein band run very slow in the gel. This might because of weak surface charge.

Incubation at 25°C would promote GsiB to form another band with molecular weight of about 110 kDa in SDS-PAGE (Figure 2(b)). In the meantime, binding GSH or GSSG would obviously reduce the top band ratio (Figure 2(b)). The top band might be dimer of GsiB, which was not separated by denature at 95°C for 3 min. To verify this conjecture, a 6×His tag was added at the C terminal of GsiB (primer GsiB-F and GsiB-6His-R) (Table 1). Using anti-6×His antibody, western blot showed both the two bands were GsiB protein (Figure 2(c)).

The results indicated that GsiB could bind both GSH and GSSG. Binding with substrate would induce conformational change of GsiB.

### 3.3. Protein Interaction of GsiB with Other Components

**Figure 3:**
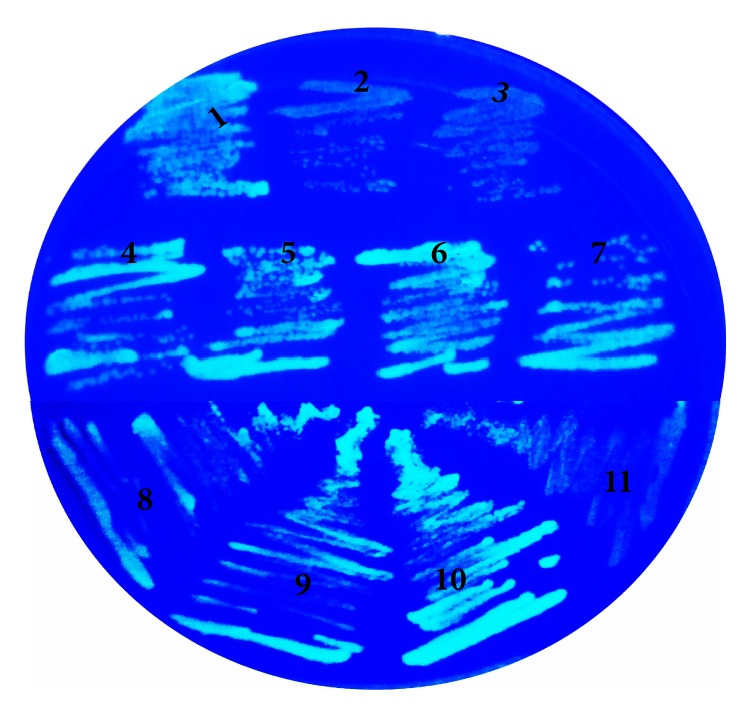
In vivo analysis of GsiB interaction with other proteins of GSI. The GSI genes in pET11a-link-NGFP and pMRBAD-link-CGFP vectors were refered to as pN- and pC-. pN-Z and pC-Z were positive control plasmids. The protein interaction was analyzed under UV light. Numbers 1 to 7 were transformants harboring: pN-Z and pC-Z, pN-gsiB and pC-gsiC, pN-gsiB and pC-gsiD, pN-gsiA and pC-gsiC, pN-gsiA and pC-gsiD, pN-gsiC and pC-gsiD, and pN-gsiC and pC-gsiA on LB plate. To characterize function of glutathione in protein interaction, M9 medium plate was used with GSH as sole sulfur source. Number 8 to 11 were transformants harboring: pN-Z and pC-Z, pN-gsiB and pC-gsiC, pN-gsiB and pC-gsiD, and pN-gsiB and pC-A.

Interaction of GsiB with other components of GSI was determined. pET11a-link-NGFP carrying* GsiA*,* GsiB*,* GsiC,* and pMRBAD-link-CGFP carrying* GsiA*,* GsiB*,* GsiC*, and* GsiD* were pairwise and simultaneously transformed into BL21(DE3). 10 *μ*M IPTG and 0.2% arabinose were added for inducing protein expression, which made GFP reassembly possible. The reassembled GFP would show fluorescent* in-vivo*, especially under UV light (Figure 3).

The results showed that GsiB could not interact with the other three proteins on LB plate. Without binding glutathione, GsiB might present in inactive conformation. It was speculated that glutathione binding might promote conformational change, which would facilitate GsiB to interact with other components. To verify this hypothesis, M9 medium plate with glutathione as sole sulfur source was made and the interaction was characterized. As shown in Figure 3, GsiB could interact with transmembrane proteins GsiC and GsiD. However, GsiB showed no interaction with GsiA. It might be associated with their different cell locations. GsiA and GsiB were predicted to be located in the cytoplasm and periplasm of cell, respectively [13].

### 3.4. GsiB Was Essential for GSI Mediated Glutathione Import

The* GsiB* and* ggt* gene in* E. coli *were replaced by kanamycin and Chloramphenicol resistant gene. The gene deletion strains were named* ∆GsiB*,* ∆ggt*, and* ∆GsiB∆ggt*. The deletion was verified by PCR.

**Figure 4:**
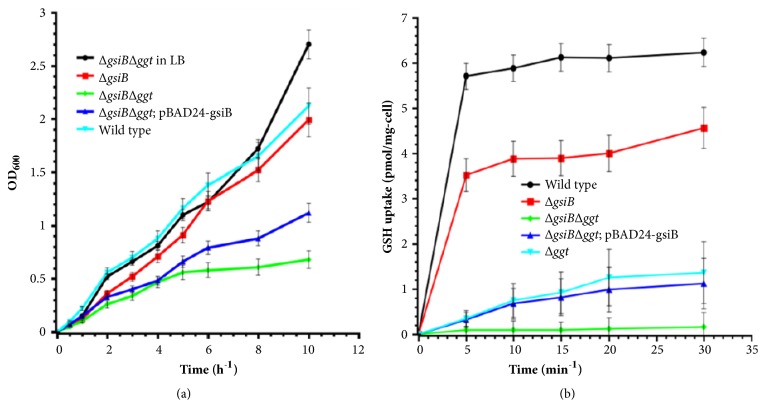
Effects of* GsiB* deletion on cell growth and glutathione uptake. (a)* GsiB* and* ggt* gene deletion strains were constructed. The growth curves of mutant and wild type* E. coli* were recorded. pBAD24-gsiB was transformed into* ΔGsiBΔggt* to compensate for gene defection. (b) The effects of GsiB on glutathione import was determineted by recording glutathione concentration change in the medium, which was measured by Glutathione Assay Kit (Sigma). The glutathione uptake curves of mutant and wild type* E. coli* were analyzed.

The cell growth and glutathione uptake curves were measured, using M9 medium with glutathione as sole sulfur source. The results suggested that* ∆GsiB∆ggt *strain grew much slower in glutathione containing M9 medium than in LB medium. The slow growth rate could be somewhat compensated by transformation of pBAD24-gsiB (Figure 4(a)).* ∆GsiB *grew faster than* ∆GsiB∆ggt *with or without pBAD24-gsiB.

*∆ggt *could uptake glutathione at a lower rate than wild type and* ∆GsiB*. However, the glutathione import in* ∆GsiB∆ggt *strain was undetectable (Figure 4(b)). The results depicted that GGT pathway was more effective, which might mediate more glutathione uptake than GSI. As* GsiB* gene deletion could block GSI mediated glutathione uptake, which was compensated by pBAD24-gsiB (Figure 4(b)). GsiB was essential for GSI mediated glutathione uptake.

## 4. Discussion

Glutathione is the most abundant small molecular weight thiol containing antioxidant in living cells and plays a plethora of cellular roles. GsiB is the glutathione binding protein of GSI, which specifies the transporter. Putting deep insights into functions of GsiB will help to elucidate the mechanism of specific glutathione import.

The Ni^2+^-NTA column could enrich His-tagged GsiB, comprising more than 90% of total proteins. MBP was used as fusion protein which can assist protein folding. The MBP fusion can be removed by MBP column. MBP used here promoted solubility of GsiB. By using different tags and purification columns, the purification of proteins could be efficient.

Lower inducing temperature and IPTG concentration would contribute to soluble expression of GsiB. Induced at 22°C with 0.1 mM IPTG, GsiB was expressed mainly in soluble fraction. Protein expression was confirmed by Western blot. High yield of pure GsiB protein will contribute to future biophysical and biochemical studies.

The freshly purified GsiB showed only one band in SDS-PAGE and two bands in native gel. GsiB might be present as monomer and dimer here. However, after incubation at 25°C, another protein band appeared in SDS-PAGE. This band might be dimer of GsiB, as the molecular weight was about 110 kDa. This top protein band was stable, which would not be separated by denature at 95°C. However, the top band could be reduced by incubation with GSH or GSSG. As shown by crystal structure (PDB ID: 1UQW), the N-terminal fragment was located at surface of GsiB. Although not included in the structure, Cys23 was speculated to locate at the surface of full length GsiB, which might form disulfide bond between proteins. The band could be disrupted by glutathione. Or the binding of GSH or GSSG could block Cys23 site, which probably affect disulfide bridge formation. In the meantime, crystal structure of GsiB was shown to have A and B chains. Chain A and B both contained a GsiB protein. The two chains of GsiB protein had different structures, which was in consistence with two conformations in native gel.

GsiB didn't interact with the other three proteins of GSI on LB plate. However, GsiB could interact with the inner-membrane proteins GsiC and GsiD when glutathione was used as sole sulfur source. It was speculated that GSI had two state: ‘open' and ‘close'. The state of GSI depends mainly on GsiB protein conformation. Without binding glutathione, GsiB would present in ‘inactive' conformation and will not interact with GsiC or GsiD. GSI would be at ‘close' state. Binding with glutathione would change GsiB to ‘active' conformation, which facilitate GsiB to interact with inner-membrane channel. GsiA could hydrolyze ATP to support glutathion import and GSI would ‘open'. The ‘open' state of GSI required GsiB binding with glutathione and GsiA, B, C, D to interact with each other. In summary, binding with GSH or GSSG would change GsiB protein conformation from ‘inactive' to ‘active'. The activated GsiB interacted with GsiC and GsiD and substrate was then transferred into inner-membrane channel. The transportation of GSH and GSSG was powered by GsiA hydrolyzing ATP. After glutathione import, GsiB was released from the complex and wait for another transportation.

*GisB* was deleted in* E. coli* to determine the* in-vivo* function. The growth of* ∆GsiB *was not affected when using glutathione as sole sulfur source. This is because the strain could uptake glutathione from the medium by GGT pathway. Figure 4(b) showed that GGT pathway could mediate much more glutathione import than GSI. Glutathione imported by GGT was then hydrolyzed to glutamic acid and cysteinylglycine [21]. Cysteinylglycine was cleaved into cysteine and glycine by aminopeptidases A, B, and N and dipeptidase D. So glutathione could serve as sulfur source for* ∆GsiB* to survive and grow. However, the growth of* ∆GsiB∆ggt* was affected with glutathione as sole sulfur source. As shown in Figure 4(b), the glutathione uptake by* ∆GsiB∆ggt *strain was undetectable. The glutathione import was compensated by transformation of pBAD24-gsiB. The results showed that GsiB was essential for GSI mediated glutathione import.

Collectively, the glutathione binding protein GsiB from* E. coli* was expressed and characterized. Investigation of biological functions and protein interactions of GsiB would help to elucidate the specific glutathione import mechanism.

## Figures and Tables

**Table 1:** Primers for gene expression, protein interaction and gene deletion.

Primer	Sequence 5‘ - 3'
GsiB-F	CATG CC ATGG CAAGAGCTGTACACCGTAG
GsiB-R	CCC AAGCTT ATTGCAAATCCGCGTCTTC
N-GsiA-F	CCATCTCGAG GCCACACAGTGATGAACTTGATG
N-GsiA-R	CGTCGGATCC TTATCTACGCATGAATGCGTATTCT
N-GsiB-F	CCATCTCGAG GGCAAGAGCTGTACACCGTAGTG
N-GsiB-R	CCATCTCGAG TTATTGCAAATCCGCGTCTTC
N-GsiC-F	CCATCTCGAG GCTTAATTACGTTATCAAACGCTTA
N-GsiC-R	CGTCGGATCC TTACTTGTACCTGATAGCCGGGTTA
C-GsiA-F	CATG CCATGG TGCCACACAGTGATGAACTTGATG
C-GsiA-R	CATGGACGTC CC TCTACGCATGAATGCGTATTCTG
C-GsiB-F	CATG CCATGG CAAGAGCTGTACACCGTAGTG
C-GsiB-R	CATGGACGTC CC TTGCAAATCCGCGTCTTCAAAG
C-GsiC-F	CATG CCATGG TGCTTAATTACGTTATCAAACGCT
C-GsiC-R	CATGGACGTC CC CTTGTACCTGATAGCCGGGTTA
C-GsiD-F	CATG CCATGG TG CGACTATTTAACTGGCGACG
C-GsiD-R	CATG CCATGG CC TCCTTTAATTTTCGGATCCAGC
GsiB-Del-F	GCATTACGTCGCACAACCACAATCAGAATACGCATTCATGCGTAGATAACATTCAGGC GTGTAGGCTGGAGCTGCTTC
GsiB-Del-R	AACAGCGTCGGAATCAACCCCAGTAAGCGTTTGATAACGTAATTAAGCATTCCACTCC CATATGAATATCCTCCTTAG
GGT-Del-F	CGATGATTAATTCAGAGTTATATACCAGGCTTAGCTGGGGTTGCCCCTTAATCTCTGGAG GTGTAGGCTGGAGCTGCTTC
GGT-Del-R	AGGCTACCTTCGGCTTGCCCTGACAAAATAGCCCTCTTCCCACGAAGAGGGCCGCTAACC CATATGAATATCCTCCTTAG
Del-F	GTGTAGGCTGGAGCTGCTTC
GsiB-Del-R	ACACCAGCACCGAGACGA
GGT-Del-R	GAACGGCAAAACCGCTGGA
GsiB-6His-R	CCC AAGCTT ACATCACCATCACCATCACTTGCAAATCCGCGT CTTCA

## Data Availability

The glutathione import related data used to support the findings of this study are included within the article. The information of plasmids used in this study is available from the corresponding author upon request.
